# Prediction of the potentially suitable areas of *Eucommia ulmoides* Oliver in China under climate change based on optimized Biomod2 and MaxEnt models

**DOI:** 10.3389/fpls.2024.1359271

**Published:** 2024-11-15

**Authors:** Guoqiong Cao, Xiaofeng Yuan, Qilin Shu, Yayang Gao, Taosheng Wu, Chenghong Xiao, Jian Xu, Yongping Zhang

**Affiliations:** ^1^ National Engineering Technology Research Center for Miao Medicine, Guizhou Engineering Technology Research Center for Processing and Preparation of Traditional Chinese Medicine and Ethnic Medicine, College of Pharmaceutical Sciences, Guizhou University of Traditional Chinese Medicine, Guiyang, China; ^2^ Resource Institute for Chinese & Ethnic Materia Medica, Guizhou University of Traditional Chinese Medicine, Guiyang, China

**Keywords:** climate change, potentially suitable area, species distribution models, woody medicinal plant, MaxEnt

## Abstract

*Eucommia ulmoides* Oliver is a medicinal plant of significant economic importance. Its cortex has been employed for centuries to alleviate various conditions such as lumbar pain, knee pain, and osteoporosis. Additionally, *E. ulmoides* possesses substantial industrial value. With the growing demand for this medicinal herb, ensuring its sustainable supply has become imperative. Climate change has caused habitat restrictions or migration of medicinal plants. Therefore, predicting the impact of climate change on the distribution of *E. ulmoides* is crucial for its conservation and sustainable use. This study evaluated the potential distribution of *E. ulmoides* across China under various climate change scenarios since the last interglacial period by modeling suitable areas based on 257 distribution records and 19 major environmental factors related to *E. ulmoides*. The model selection process initially identified the MaxEnt model as the most suitable. The optimized MaxEnt model, with RM = 2.0 and FC = LQHPT settings, generated the most precise predictions. Results indicate that the minimum temperature of the coldest month, annual mean temperature, and annual precipitation significantly affect the distribution of *E. ulmoides*. Under current environmental conditions, highly suitable areas for *E. ulmoides* are found in Southwest and Southeast China, with a total suitable habitat area of 23.12 × 10^4^ km^2^. However, the range of suitable habitat has shifted due to global warming’s negative impact. Under different climate scenarios, suitable areas for *E. ulmoides* have either increased or decreased, with expansions primarily in high-latitude regions. Future climate scenarios predict shifts in the centroid of suitable *E. ulmoides* habitat towards Yichang City in Hubei Province. The findings of this study support the development, artificial cultivation, and conservation of *E. ulmoides* resources.

## 1 Introduction

The modification of ecological habitats and geographical distribution of plants is substantially influenced by global climate change (Li et al., 2020b; Wouyou et al., 2022; Zhang K. et al., 2019; Zhang, Y. et al., 2019). Climate change is anticipated to increasingly impact species distributions in the future (Thuiller et al., 2005). Many plant species are expected to adjust their suitable habitats in response to changing environmental conditions, potentially shifting to higher elevations or moving northward in the Northern Hemisphere (Wang et al., 2019a, b). Moreover, climate change affects sensitive ecological responses of plants, such as the duration of growing seasons and flowering periods (Hampe et al., 2013; Wang et al., 2013). Extensive research indicates that climate change has profound effects on ecosystem functions and global biodiversity (Li et al., 2019; Watson et al., 2017). Predicting how climate change will impact past and current species habitats is crucial for understanding potential responses to future environmental changes.

Modeling the impacts of climate change on species’ habitats across landscapes, both historically and currently, is essential for comprehending potential future responses (Zhang K. et al., 2019; Deb et al., 2017; Zhao et al., 2021a). Advances in technology now make it possible to predict relationships between species distributions and various environmental factors, including climate, topography, meteorological data, and species data (Zhao et al., 2022). In research on how climate change affects plant geographical distribution, niche models based on species distributions have become a significant trend (Warren et al., 2010). Ecological niche models are increasingly employed in studies of biological responses to climate change, biological invasion, conservation biology, and various aspects of ecology and evolutionary biology (Sillero et al., 2020; Ferreira et al., 2022; Feng et al., 2019). In recent decades, numerous models, such as the generalized linear (GLM) (Hirzel et al., 2001), maximum entropy (MaxEnt) (Phillips et al., 2006), and the generalized additive model (GAM) (Austin et al., 2009), have been developed and employed for predicting suitable plant areas. For example, the use of MaxEnt to predict the impact of climate change on *Sinopodophyllum hexandrum* (Lai et al., 2022), *Paris verticillata* (Ji et al., 2020), and *Ophiocordyceps sinensis* (Wei Y. et al., 2021). Each model has its own strengths and limitations due to differences in principles and algorithms. It is noteworthy that many previous studies have directly used one model to study the effects of climate change on species distribution and planning of protected areas, without taking into account that different modeling techniques for the same species may yield different results. Biomod is an R-based modeling platform designed to enhance prediction accuracy. Biomod offers ten commonly used species distribution models. Users can freely select models from this platform while customizing them by setting conditions to achieve optimal prediction outcomes.

*Eucommia ulmoides* Oliver (*E. ulmoides*), also known as Du Zhong, is a deciduous tree from the unique genus Eucommia. Although *E. ulmoides* was historically widespread across the Northern Hemisphere, it became extinct in North America and other parts of Europe during the Quaternary Ice Age, surviving only in China (Huang et al., 2021; Wu et al., 2018). Native to China, *E. ulmoides* is categorized as a rare and endangered species (Wang et al., 2017). Its bark has been highly esteemed as a medicinal resource for over 2000 years and is a key component in traditional Chinese medicine (TCM) formulations (Zhu et al., 2016). In addition to its historical uses for liver and kidney nourishment, bone and tendon strengthening, and fetal calming, recent studies have highlighted the medicinal value of its leaves, bark, male flowers, and fruits (Zhu and Sun, 2018). Furthermore, the gum from *E. ulmoides* is utilized in various industries, including transportation, sports, aerospace engineering, and construction (Li et al., 2022; Zhang et al., 2011).

Previous studies on *E. ulmoides* have primarily focused on its pharmacological actions and chemical composition, while the understanding of its biology and potential suitable habitats remains limited. Future climate change may influence the survival, replacement, and community dynamics of *E. ulmoides*. To enhance our understanding of how climate change impacts the suitable distribution range of *E. ulmoides*, it is crucial for effectively conserving its germplasm resources and promoting sustainable cultivation practices. Some researchers have used the MaxEnt model to identify the suitable area of *E. ulmoides* in Ruyang County (Wang J. et al., 2023), but the geographical distribution trend of *E. ulmoides* in China based on future climate change has not been reported.

This study is first to use Biomod2 to screen ecological models and optimize them to predict the potential suitable planting area of *E. ulmoides* in China under climate change. The objectives of this study include to (1) compare the accuracy of *E. ulmoides* prediction by nine models provided by Biomod2, (2) predict the distribution of potentially suitable areas for *E. ulmoides* in China under current climate conditions, and classify these areas into different suitability categories, (3) identify the environmental factors that restrict the potential geographical distribution of *E. ulmoides*, and (4) forecast the changes in potentially suitable areas under various future climate scenarios. The focus was on analyzing how the potential geographical distribution of *E. ulmoides* has been influenced by climate change over different periods. The results offer valuable insights for the introduction, cultivation, conservation, and effective use of *E. ulmoides*.

## 2 Materials and methods

### 2.1 Study area

*E. ulmoides* Oliver, the sole extant species of Eucommiaceae, is an endemic dioecious tree in China (Zhang et al., 2016). The current geographical distribution of *E. ulmoides* in China results from its artificial introduction from its central origin to various regions across the country. The distribution area of *E. ulmoides* is relatively extensive, covering the hills and mountains in the plains of Gansu, Shaanxi, Guizhou, Hubei, Hunan, Henan, Sichuan, Jiangxi, Anhui, Zhejiang, Shanxi, Fujian, Guangxi, Guangdong, Jiangsu, and Henan. The primary distribution area of *E. ulmoides* is located between 98–123°E and 18–42°N.

### 2.2 Occurrence data

**Figure 1:**
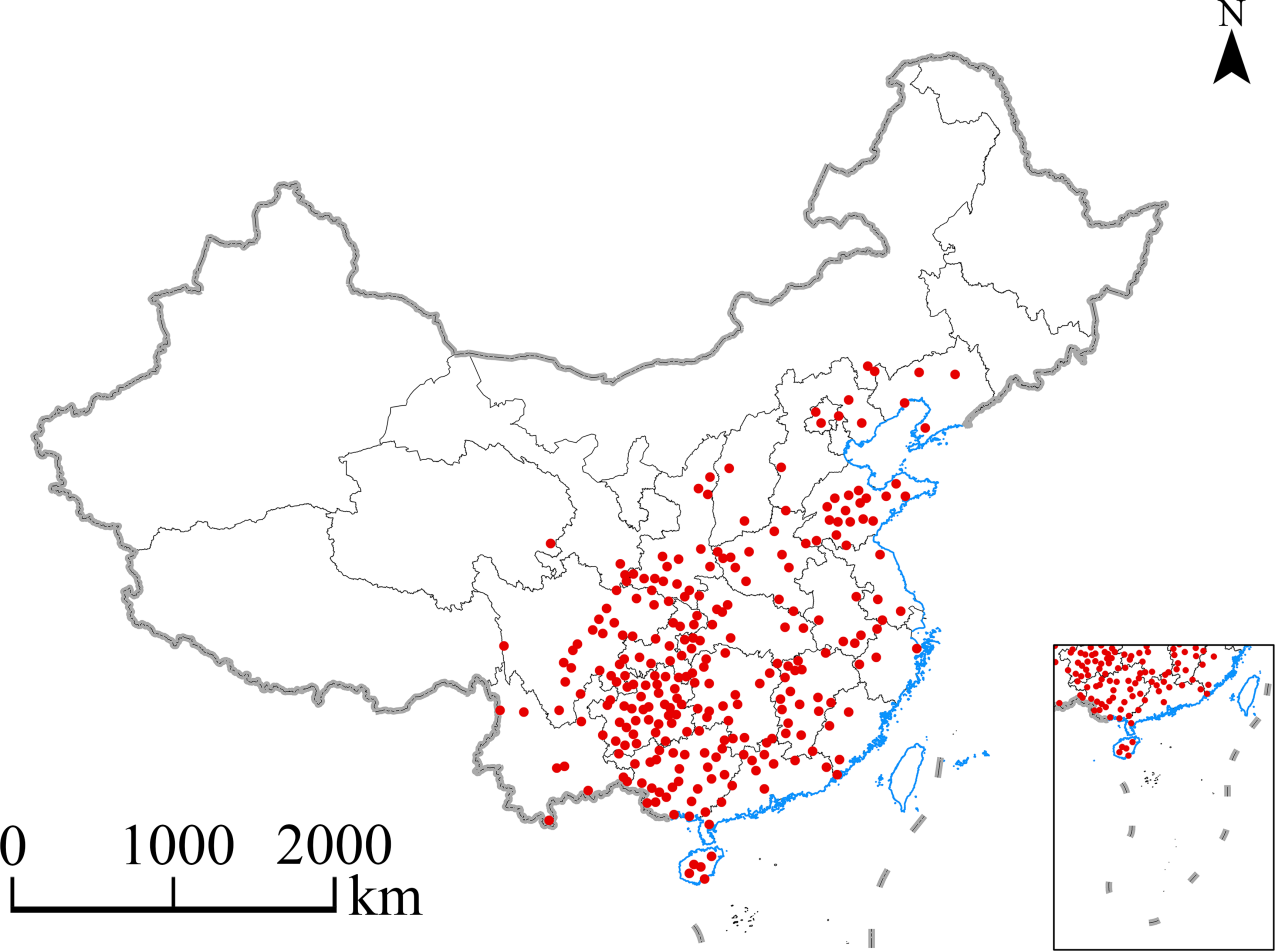
Distributions of occurrence points of *E. ulmoides* in China.

A total of 957 distribution records were gathered through a review of published documents and searches in various databases, including the China Digital Herbarium (http://www.cvh.org.cn), the Chinese Plant Image Database (http://ppbc.iplant.cn/special), the National Specimen Information Infrastructure (NSII, http://www.nsii.org.cn/), and other herbaria, between December 2022 and February 2023. The R software package “spThin” was utilized to reduce sampling bias to remove records clustered within a 10 km × 10 km area (Zhang et al., 2018). Ultimately, 257 points were selected for further analysis (**Supplementary Table S1**). The primary areas where *E. ulmoides* was found were determined based on the distribution data (**Figure 1**). The geographical base map of China used was a 1:400,000 vector map showing administrative divisions, downloaded from the National Basic Geographic Information System website (https://nfgis.nsdi.gov.cn).

### 2.3 Environmental variables

Climate data for various periods (the last interglacial period, the last glacial maximum, the mid-Holocene, the modern era, and the future) were obtained from the World Climate Database (http://worldclim.org) in March 2023. All environmental data were set to a spatial resolution of 30 arcseconds by ArcGIS software. For the analysis of past climate data, three specific periods were selected: the last interglacial period (LIG 130 ka BP), the last glacial maximum (LGM 21 ka BP), and the mid-Holocene (MH 6 ka BP). Future climate data were sourced from the Beijing Climate Center Climate System Model (BCC-CSM2-MR) of the Coupled Model Intercomparison Project Phase 6 (CMIP6) (Wu et al., 2019), covering four periods: 2021–2040 (2030s), 2041–2060 (2050s), 2061–2080 (2070s), and 2081–2100 (2090s). Data were analyzed under three shared socioeconomic pathways (SSP1-2.6, SSP2-4.5, and SSP5-8.5). **Supplementary Table S2** provides information on the 19 bioclimatic variables. Since topographic and soil variables are not expected to undergo significant changes, this study focuses solely on predicting the impact of climate on the suitable habitat for *E. ulmoides.* The environmental variables were screened (Wang Y. et al., 2023) to evaluate the contribution of each ecological factor to the predicted distribution of *E. ulmoides*. First variables with cumulative contributions exceeding 95% were selected. Subsequently, Pearson correlation analysis was conducted. Among pairs of highly correlated variables (r > 0.8), the variable with the greater contribution to the response was retained. In the end, 10 major environmental variables were included in the MaxEnt simulations (**Table 1**).

**Table 1:** Percent contribution and permutation importance of screened environmental variable of *E. ulmoides* in China.

Variables	Description	Contribution (%)	Permutation importance(%)	Units
bio6	Min temperature of the coldest month	47.6	22.9	°C
bio1	Annual mean temperature	19.3	3.3	°C
bio12	Annual precipitation	12.7	1.8	mm
bio13	Precipitation of the wettest month	4.6	2.9	mm
bio4	Temperature seasonality (standard deviation×100)	2.4	6.9	–
bio11	Mean temperature of the coldest quarter	2.2	0.3	°C
bio3	Isothermality (bio2/bio7) (× 100)	2.2	2.7	–
bio2	Mean diurnal range (Mean of monthly (max.temp.-min.temp.)	1.9	0.5	°C
bio5	Max temperature of the warmest month	1.1	5.5	°C
bio8	Mean temperature of the wettest quarter	1.1	0.9	°C

### 2.4 Model calibration

Firstly, we adopted Biomod2 package to screen the models including: GLM, GBM, GAM, CTA, ANN, FDA, RF, MARS, and MaxEnt. All algorithms used the default model settings of Biomod2. In the modeling process, 75% of the 257 distribution data samples for *E. ulmoides* were randomly selected as training data, while the remaining 25% were used as testing data (5 replications) (Phillips et al., 2017). Additionally, 1000 pseudo-absence points were randomly chosen, and this process was repeated three times for model construction. In total, 135 distinct models were developed. The accuracy of the nine models’ predictions was evaluated using the area under the receiver operating characteristic (ROC) curve (AUC) and the true skill statistic (TSS). The AUC ranged from 0 to 1.0. The higher the AUC value is, the more accurate the prediction result. An AUC ranging from 0.5 to 0.7 indicates poor accuracy, 0.7 to 0.8 indicates mediocre accuracy, 0.8 to 0.9 indicates good accuracy, and 0.9 to 1.0 indicates very good accuracy (Dakhil et al., 2019). The TSS values range from 0 to 1.0, values from 0.55 to 0.7 indicate general values, and values from 0.7 to 0.8 indicate good values. After the screening, the selected model is further optimized.

The optimized values for the regularization multiplier (RM) and feature combination (FC) were determined using the ENMeval package in R. The RM values ranged from 0.5 to 4, in increments of 0.5 (totaling 8 values). Nine different FCs were tested: L, LQ, H, LQH, LQHP, LQHPT, QHP, QHPT, and HPT (Velasco and González-Salazar, 2019). The ENMeval package evaluated 72 parameter combinations and was used to calculate the corrected Akaike minimum information criterion (AIC) model. The AIC value assessed the model’s complexity and fit. The delta.AICc, testing AUC (avg.diff. AUC), and the average 10% test omission rate (avg.test.or10pct) were used to evaluate the model’s fit to the native species distribution points (Muscarella et al., 2015).

### 2.5 Changes in the spatial pattern of the suitable distribution area for *E. ulmoides*

The model results were processed using ArcGIS 10.4.1 software. The natural breaks method was applied to classify the results and create a gradient classification for suitable habitats, dividing the distribution areas of *E. ulmoides* into four categories: unsuitable (0, 0.1), less suitable (0.1, 0.25), moderately suitable (0.25, 0.5), and highly suitable (0.5, 1) (Zhan et al., 2022). An existence/nonexistence (0, 1) matrix for *E. ulmoides* under past, current, and future climate change scenarios was established. Using this matrix, changes in the suitable distribution area of *E. ulmoides* were analyzed across different climate scenarios. Area changes were calculated relative to the current suitable habitat area for *E. ulmoides*. Changes were defined as follows: a matrix value of 0→1 indicated a newly suitable area, 1→0 indicated a lost suitable area, 1→1 indicated a retained suitable area, and 0→0 indicated an unsuitable area (Ye et al., 2020).

### 2.6 Analysis of multivariate environmental similarity surface and most dissimilar variable

The environmental variables from the current potential distribution area of *E. ulmoides* served as the reference layer. A multivariate environmental similarity surface was used to assess climatic anomalies in present suitable habitats under historical and future climate change scenarios. The analysis focused on the most dissimilar variables to identify key factors influencing changes in potential geographical distribution. MESS measures the similarity between the climate of a given location at a specific time and the climate of the reference layer. It compares a set of predicted variables (V1, V2, Vi, etc.) with a set of reference points. In this context, mini and maxi denote the minimum and maximum values of the environmental variable Vi, respectively. Pi represents the value of Vi at a point P in the reference layer during a given time period, while fi is the percentage of study area points where Vi < pi (Elith et al., 2010). When fi = 0, the similarity surface is 100 (p - mini)/(max - mini); when 0 < fi ≤ 50, the similarity surface is 2fi; when 50 < fi < 100, it is 2(100 - fi); and when fi = 100, the similarity surface is 100(max - 100)/(max - mini). The value of the multivariate environmental similarity surface at point P reflects the minimum similarity among different environmental variables, known as the maximum dissimilarity variable. A negative similarity surface indicates that at least one variable value exceeds the reference point’s environmental range for that period, termed a climate anomaly point. A value of 100 denotes complete alignment between the climate environment and the reference layer, indicating normal climatic conditions. The variable MoD shows the lowest similarity or highest degree of anomaly at a point, while the multivariate environmental similarity surface denotes the minimum similarity among all variables, highlighting the most dissimilar variable with the highest anomaly. This variable may be critical in species geographic migration.

### 2.7 Analysis of the migration of the center point of the suitable area

The SDM toolbox, a Python-based GIS software package, was employed to analyze the temporal trends in changes in suitable areas and identify these regions’ geometric centers (Pierre et al., 2018). A vector file was generated to represent the overall suitable habitat for *E. ulmoides*, illustrating shifts in centroid positions and providing insights into the direction and magnitude of changes in species habitat suitability. By monitoring variations in centroid locations through different SDMs, shifts in the distribution of *E. ulmoides* across various periods and under varying climatic conditions were investigated.

## 3 Results

### 3.1 Identification of the most appropriate niche model

**Figure 2:**
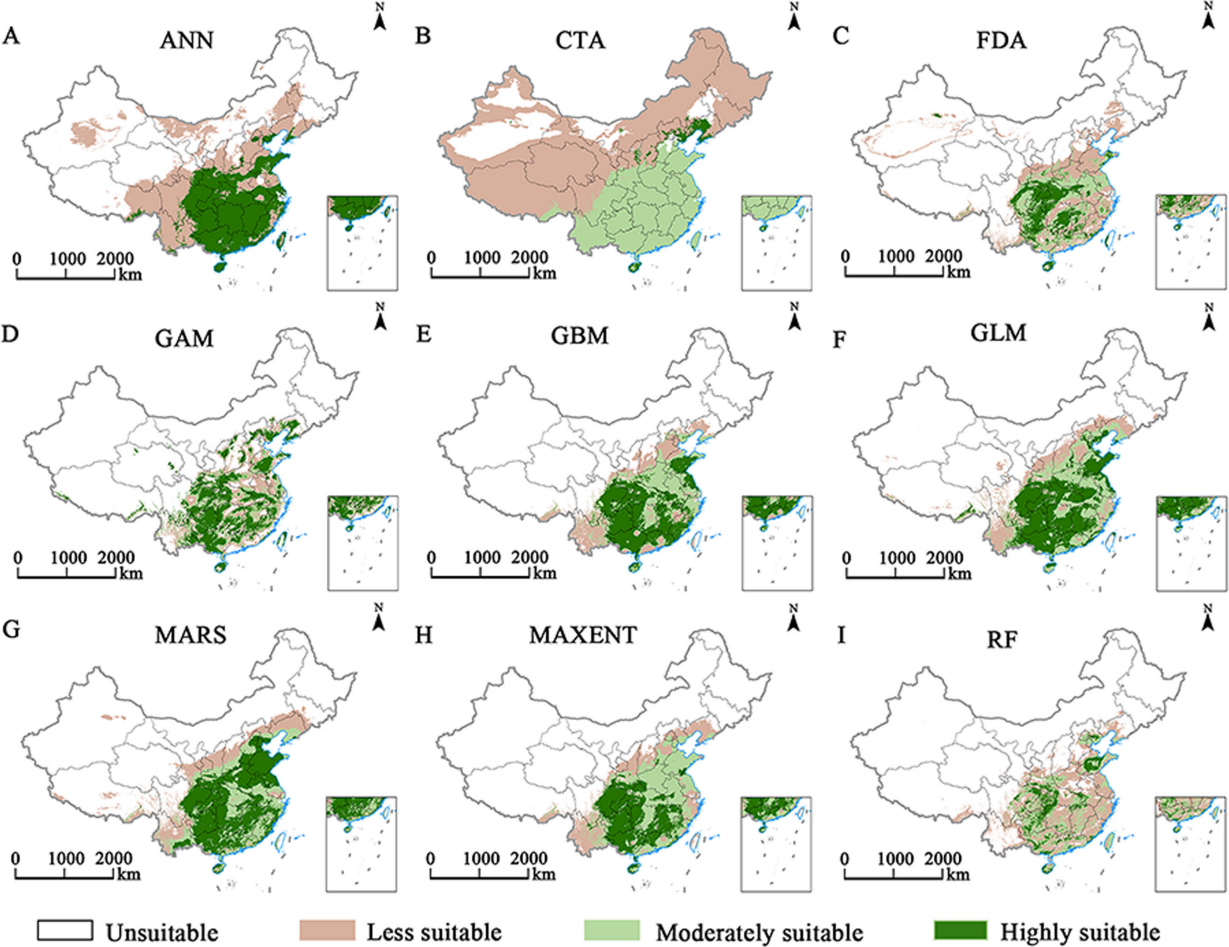
Predicted potential geographic distributions of *E. ulmoides* in China from nine spatial distribution modeling approaches **(A–I)**.

Nine niche models for species distribution were compared using the Biomod2 platform, with the MaxEnt model being identified as superior in terms of AUC and TSS. A comparison of the AUC and TSS of the 9 models revealed that the AUC and TSS of MaxEnt were significantly greater than those of the other 8 models (**Table 2**). Additionally, this model exhibited the most precise fit to the actual plant distribution (**Figure 2**); thus, it was selected for all subsequent analyses.

**Table 2:** Comparison of the *E. ulmoides* distribution in China predicted by nine niche models (n = 15).

Model	AUC	TSS
MaxEnt	0.907 ± 0.010	0.715 ± 0.018
GBM	0.891 ± 0.011**	0.685 ± 0.019**
MARS	0.890 ± 0.014**	0.654 ± 0.020**
GLM	0.890 ± 0.013**	0.644 ± 0.021**
RF	0.879 ± 0.010**	0.658 ± 0.022**
FDA	0.877 ± 0.013**	0.640 ± 0.036**
GAM	0.873 ± 0.020**	0.670 ± 0.040**
ANN	0.870 ± 0.018**	0.619 ± 0.023**
CTA	0.846 ± 0.018**	0.679 ± 0.034**

*Value is significantly different from MaxEnt, *P ≤* 0.05; **Value is significantly different from MaxEnt, *P ≤* 0.01.

### 3.2 Model accuracy evaluation

**Figure 3:**
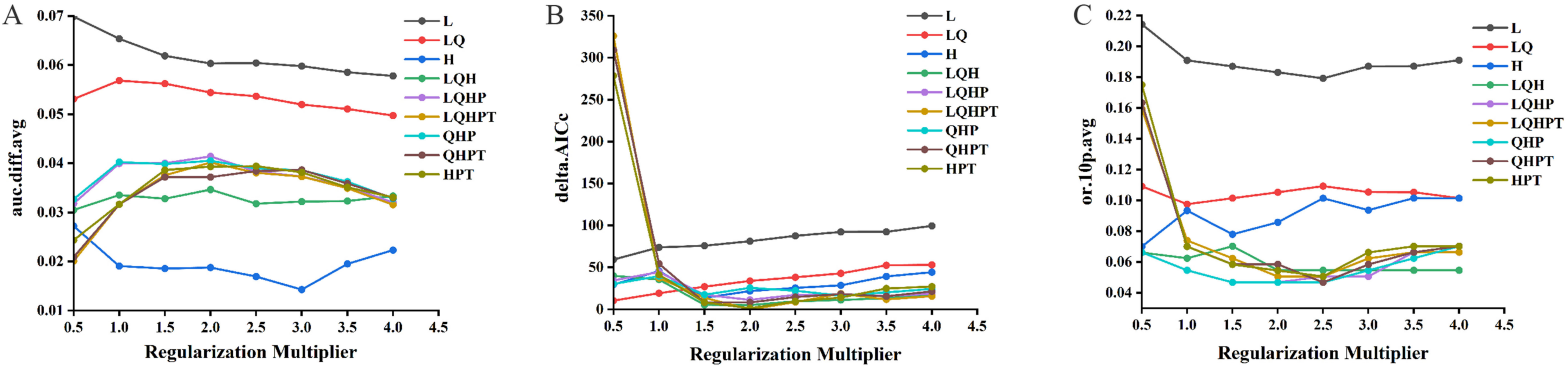
Optimization results for the MaxEnt model delta.AICc **(A)**, avg.diff. AUC **(B)** and avg.test.or10pct **(C)**.

In this study, on account of the 257 distribution points and 19 environmental variables for *E. ulmoides*, the MaxEnt model was used to simulate and predict the potential distribution of *E. ulmoides* habitats in China. Under the default parameter setting of MaxEnt, the default FC was LQHPT, the RM was 1, and the delta.AICc= = 37.48 in the MaxEnt model. When RM = 2.0, FC = LQHPT, and delta.AICc = 0, according to the AIC, the model is the optimal model (**Figure 3**). The values of avg.diff.AUC of the optimized model was 21.18% greater than that of the model with default parameters. In the optimized model, the values of avg.test.or10pct were 31.62% lower than those in the model using default parameters. The settings of the optimized parameters notably reduced the complexity and enhanced the model’s fitting degree, making it appropriate for model transfer. Consequently, RM = 2.0 and FC = LQHPT were chosen as the final parameter settings for this research.

### 3.3 Analysis of response curves

**Figure 4:**
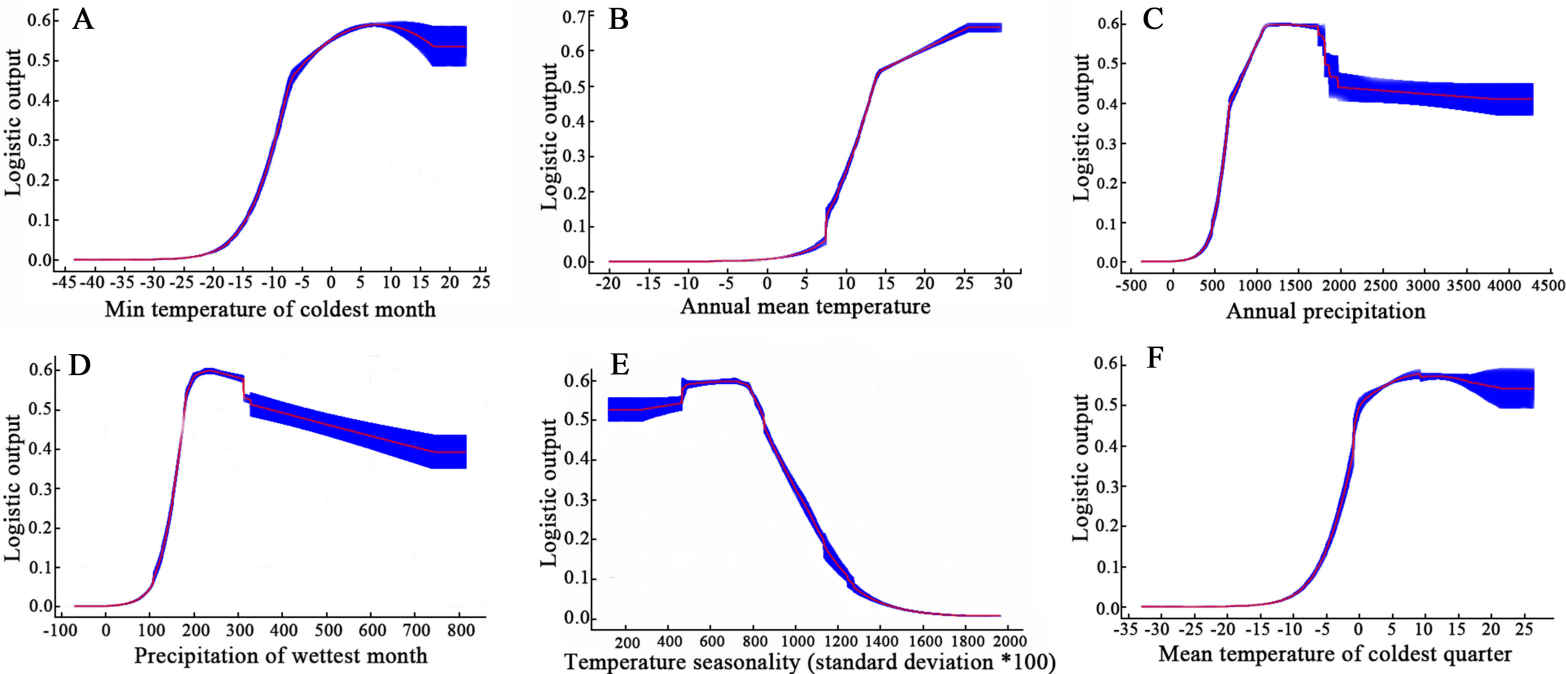
Response curve of six environmental variables in the MaxEnt model for E.ulmoides. **(A)**: Bio6; **(B)**: Bio1; **(C)**: Bio12; **(D)**: Bio13; **(E)**: Bio4; **(F)**: Bio11.

The ecological factor response curve demonstrates the influence of each environmental variable on MaxEnt predictions, showing that the fitness values produced by logistic regression vary with each environmental variable. **Figure 4** presents the response curves for the individual ecological factors affecting the distribution of *E. ulmoides*. The red segment of the curve indicates the average response from 10 repeated runs using MaxEnt software. In contrast, the blue segment shows one standard deviation above and below this average value range. Logistic regression analysis within the MaxEnt model was conducted to examine the relationships between the probability of suitable habitat distribution for *E. ulmoides* and environmental variables, producing single-factor response curves. Of the 19 environmental variables, Bio6 (minimum temperature of coldest month), Bio1 (annual mean temperature), Bio12 (annual precipitation), Bio13 (precipitation of wettest month), Bio4 (temperature seasonality), and Bio11 (mean temperature of coldest quarter) were identified as the key variables affecting the distribution of *E. ulmoides*, with contributions of 47.6%, 19.3%, and 12.7% respectively from Bio6, Bio1, and Bio12. Areas with a geographical distribution probability above 0.5 are deemed suitable habitats where the minimum temperature of the coldest month exceeds -5°C, the annual mean temperature is above 14°C, the annual precipitation ranges between 900 mm and 1900 mm, and the precipitation of the wettest month is between 190 mm and 450 mm, temperature seasonality ranges from 200 to 900, and the mean temperature during the coldest quarter surpasses 0°C.

### 3.4 Current potentially suitable habitats for *E. ulmoides*

**Figure 5:**
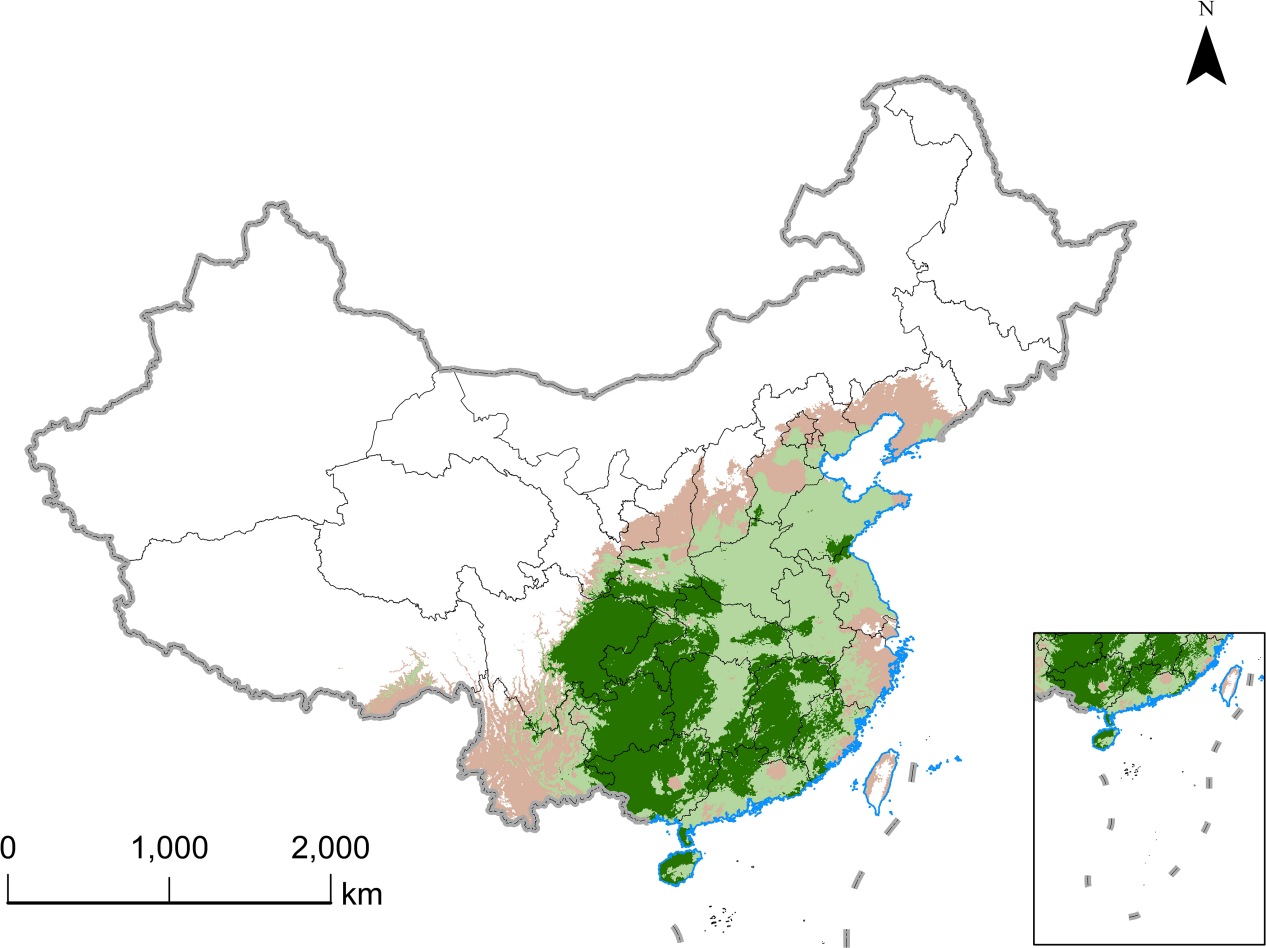
Distribution of suitable habitats for *E. ulmoides* in China under current climate scenarios predicted by MaxEnt model.

**Figure 5** illustrates the suitable area for *E. ulmoides* under current climatic conditions. According to the optimized MaxEnt model predictions, the highly suitable habitat area for *E. ulmoides* covers 10.64 × 10^4^ km^2^, while the generally suitable habitat spans 12.48 × 10^4^ km^2^, totaling 23.12 × 10^4^ km^2^. Highly suitable areas are primarily located in eastern Sichuan, Chongqing, Guizhou, Guangxi, Jiangxi, Hunan, and northern Guangdong, whereas moderately suitable areas are found in Shandong, Henan, Anhui, Jiangsu, and Hubei. The simulation and prediction outcomes align well with the geographical distribution data of *E. ulmoides*, indicating a reasonable accuracy of these results. The highly suitable areas for *E. ulmoides* in China are extensive and concentrated in growth.

### 3.5 Dynamic changes in the suitable habitats for *E. ulmoides* under different combinations of climate scenarios/years

**Figure 6:**
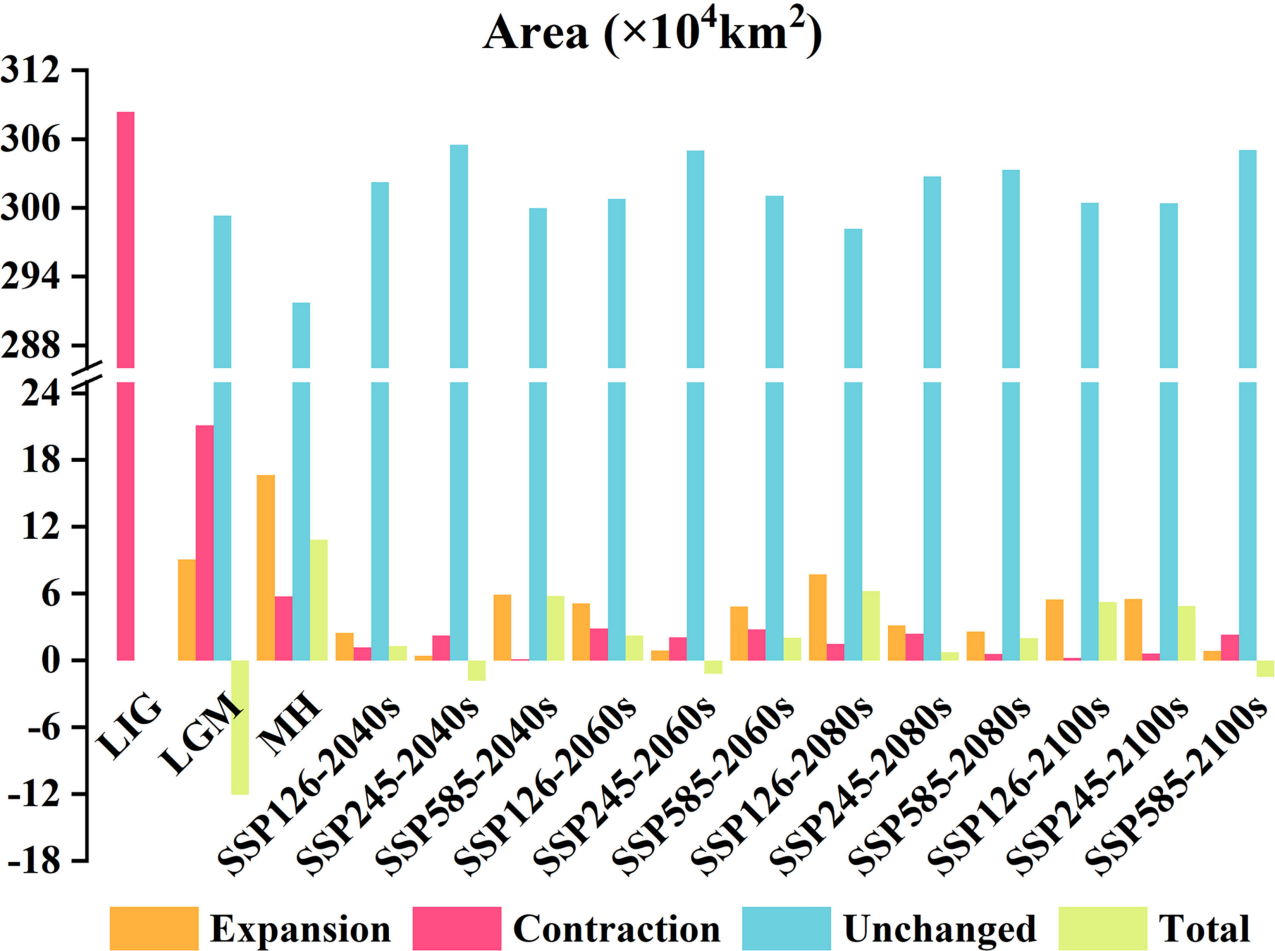
Areal change of *E. ulmoides* habitat in different periods compared with that in the current predicted by MaxEnt model.

**Figure 7:**
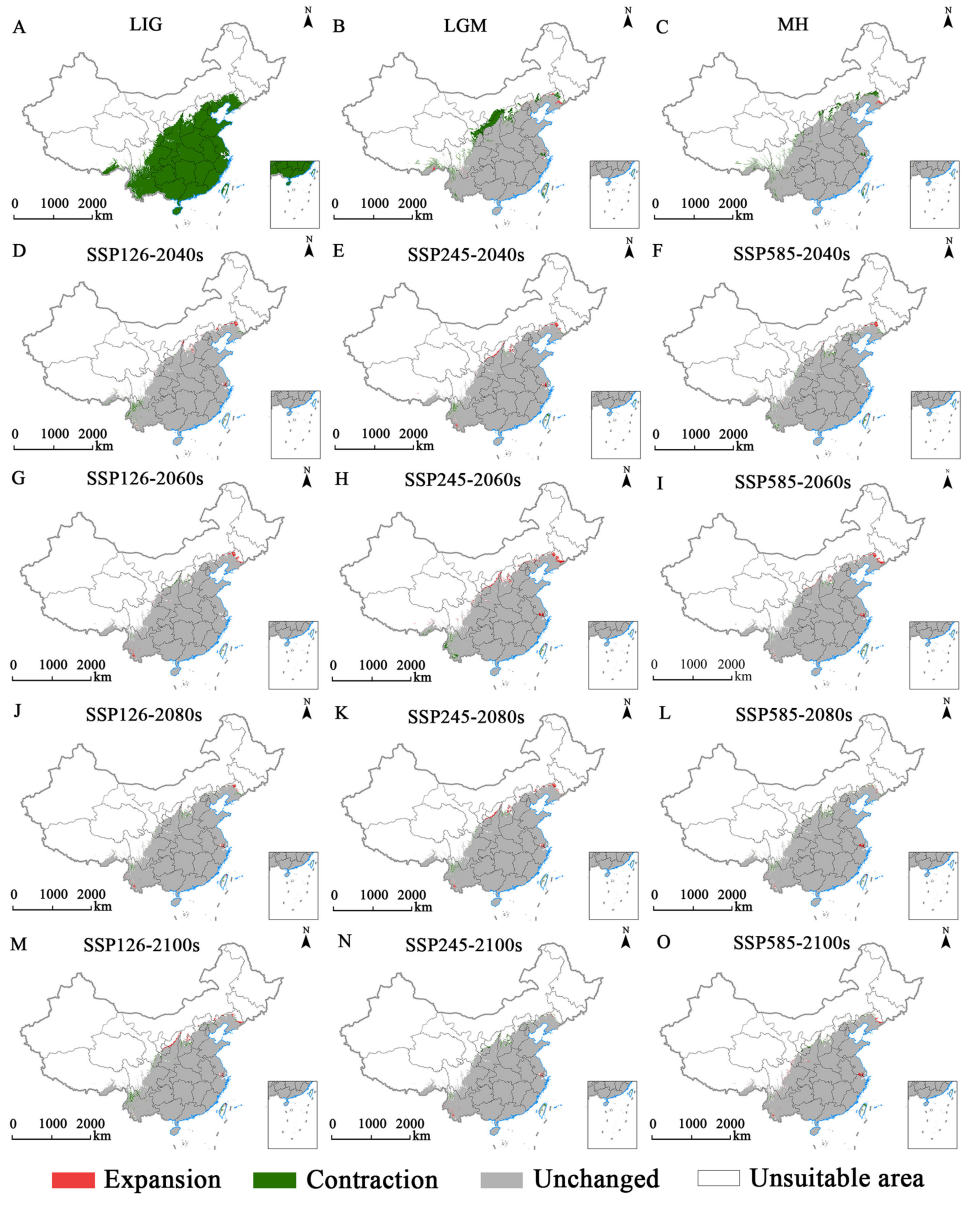
Spatial changes in *E. ulmoides* in China under the glacial epoch **(A–C)**, SSP126 **(D, G, J, M)**, SSP245 **(E, H, K, N)** and SSP585 **(F, I, L, O)** scenarios in the 2040s **(D–F)**, 2060s **(G–I)**, 2080s **(J–L)** and 2100s **(M–O)**.

A comparison of the spatial distribution of suitable *E. ulmoides* habitats during the historical period with that of modern suitable habitats revealed that **Figures 6** and **7** illustrate the changes in habitat suitability for *E. ulmoides*. During the last interglacial period, no suitable habitat was detected for *E. ulmoides*. From the last glacial maximum to the present, the suitable habitat area decreased by about 12.0663 × 10^4^ km^2^, mainly in the northern parts of the current suitable areas. Comparing the middle Holocene period to the present, the suitable habitat for *E. ulmoides* remains comparable to that of the modern era and has even slightly expanded. Additionally, an analysis of three future climate scenarios revealed that under the SSP4.5-2040s, SSP4.5-2060s, and SSP8.5-2100s projections, a decrease in suitable habitats ranging from 1.4495 × 10^4^ km^2^ to 1.8158 × 10^4^ km^2^ compared to current conditions is expected; however, aside from the mentioned above (SSP4.5-2040s, SSP4.5-2060s, and SSP8.5-2100s), all other future climate scenarios predict an increase in suitable habitats for *E. ulmoides* with an expanded range varying from 0.7534 × 10^4^ km^2^ to 6.2184 × 10^4^ km^2^.

### 3.6 Multivariate environment similarity surface and most dissimilar variable analysis

**Figure 8:**
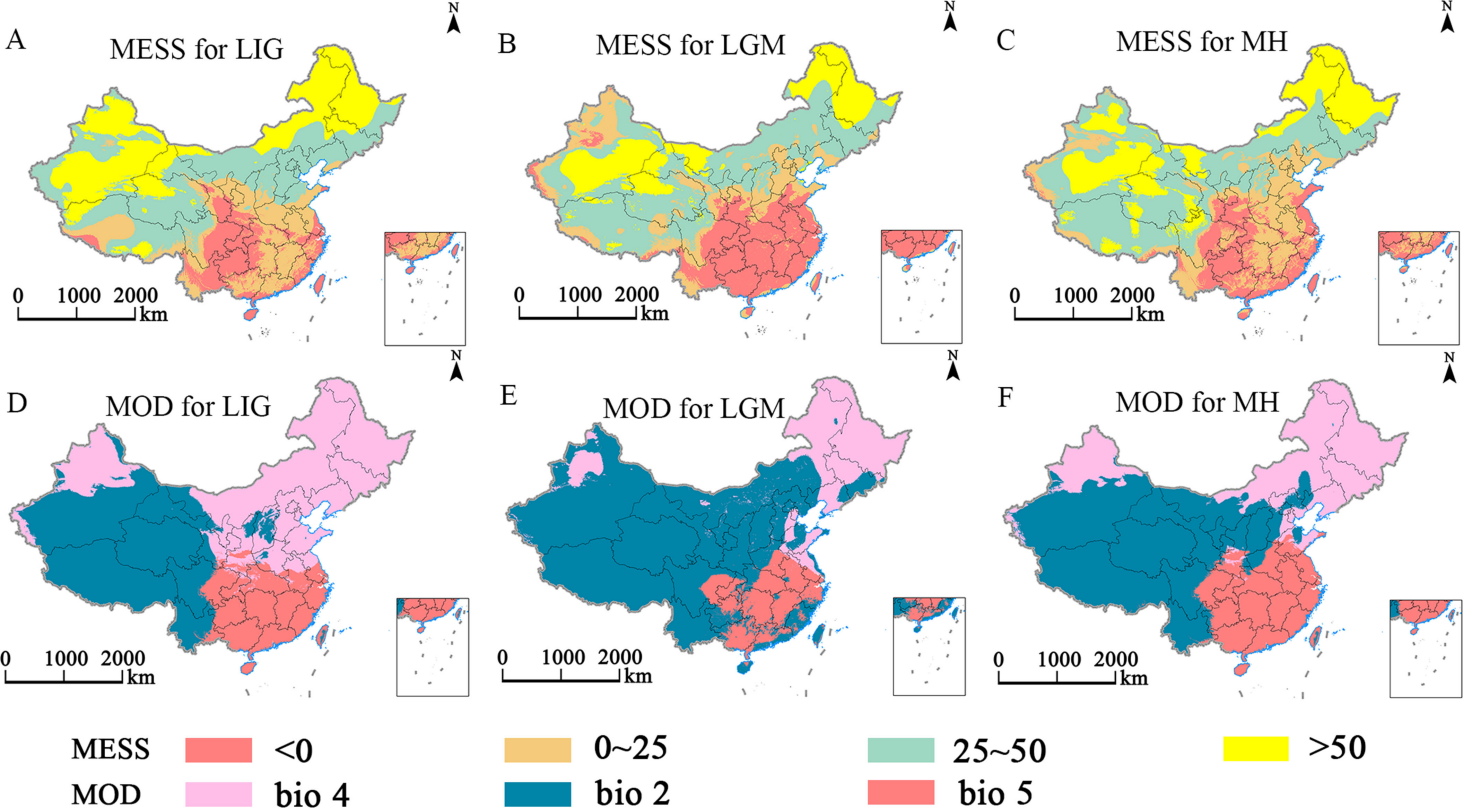
Multivariate environmental similarity surface (MESS, **A–C**) and most dissimilar variable (MoD, **D–F**) analysis for *E. ulmoides* during glacial periods.

**Figure 9:**
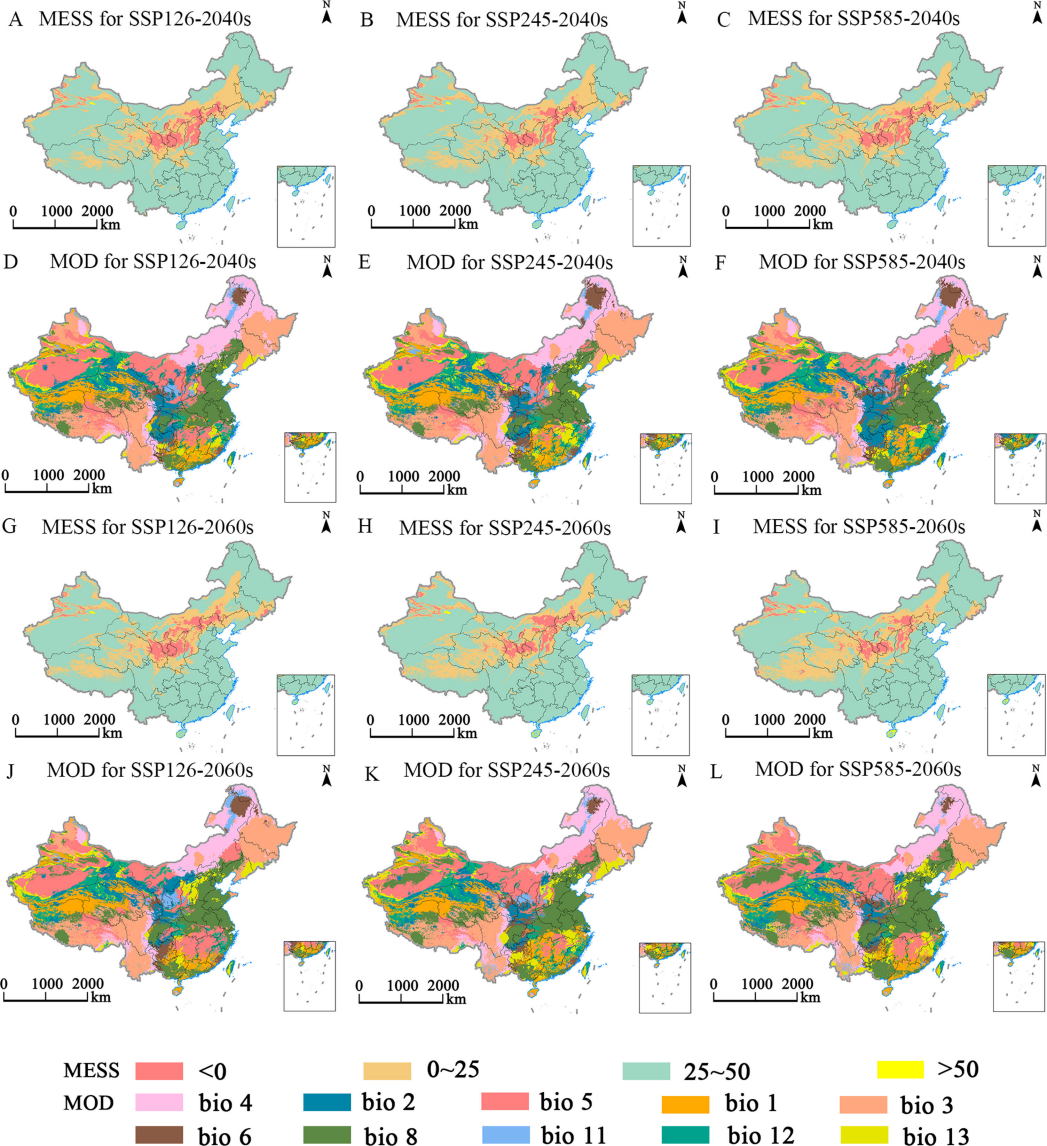
Multivariate environmental similarity surface (MESS, **A-C, G-I**) and most dissimilar (MoD, **D-F, J-L**) variable analysis for *E. ulmoides* in China under different combinations of climate change scenario in the 2040s and 2060s.

**Figure 10:**
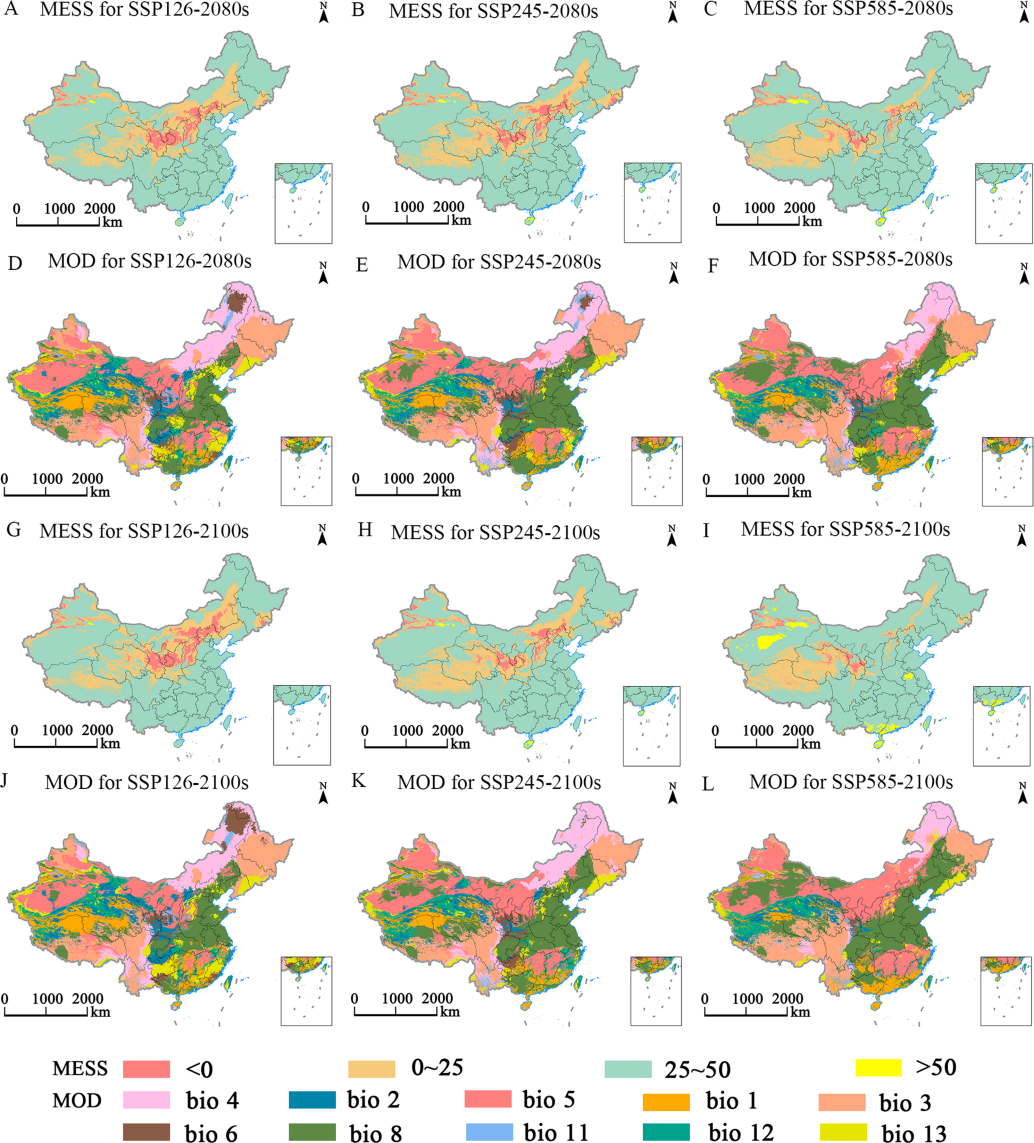
Multivariate environmental similarity surface (MESS, **A-C, G-I**) and most dissimilar (MoD, **D-F, J-L**) variable analysis for *E. ulmoides* in China under different combinations of climate change scenarios in the 2080s and 2100s.

The MaxEnt model was employed to predict the multiple environmental similarity surface (MESS) and the least similar variable (MOD) outcomes for *E. ulmoides* under different climatic scenarios (**Figures 8**–**10**). This study revealed that during the LGM, LIG, and MH, the areas of climatic anomaly (S<0) were more extensive than those projected under future climate scenarios. The historical periods were categorized by decreasing degrees of climatic anomalies in the order: LGM > MH > LIG. The primary regions of climatic anomalies were situated within the current suitable habitats. Moreover, a shift in the dominant variable affected the distribution from the mean diurnal range (bio2) to the maximum temperature of the warmest month (bio5). In contrast, under future climate scenarios, a reduction in the overall climatic anomaly area (S<0) across the potential distribution range is expected. However, changes in the severity of these anomalies are anticipated to be minor. The main regions experiencing climatic anomalies are projected to be in the northern and northwestern parts of the current suitable habitats.

### 3.7 The migration trends of the geometric centers of suitable *E. ulmoides* habitat under climate change during different periods

**Figure 11:**
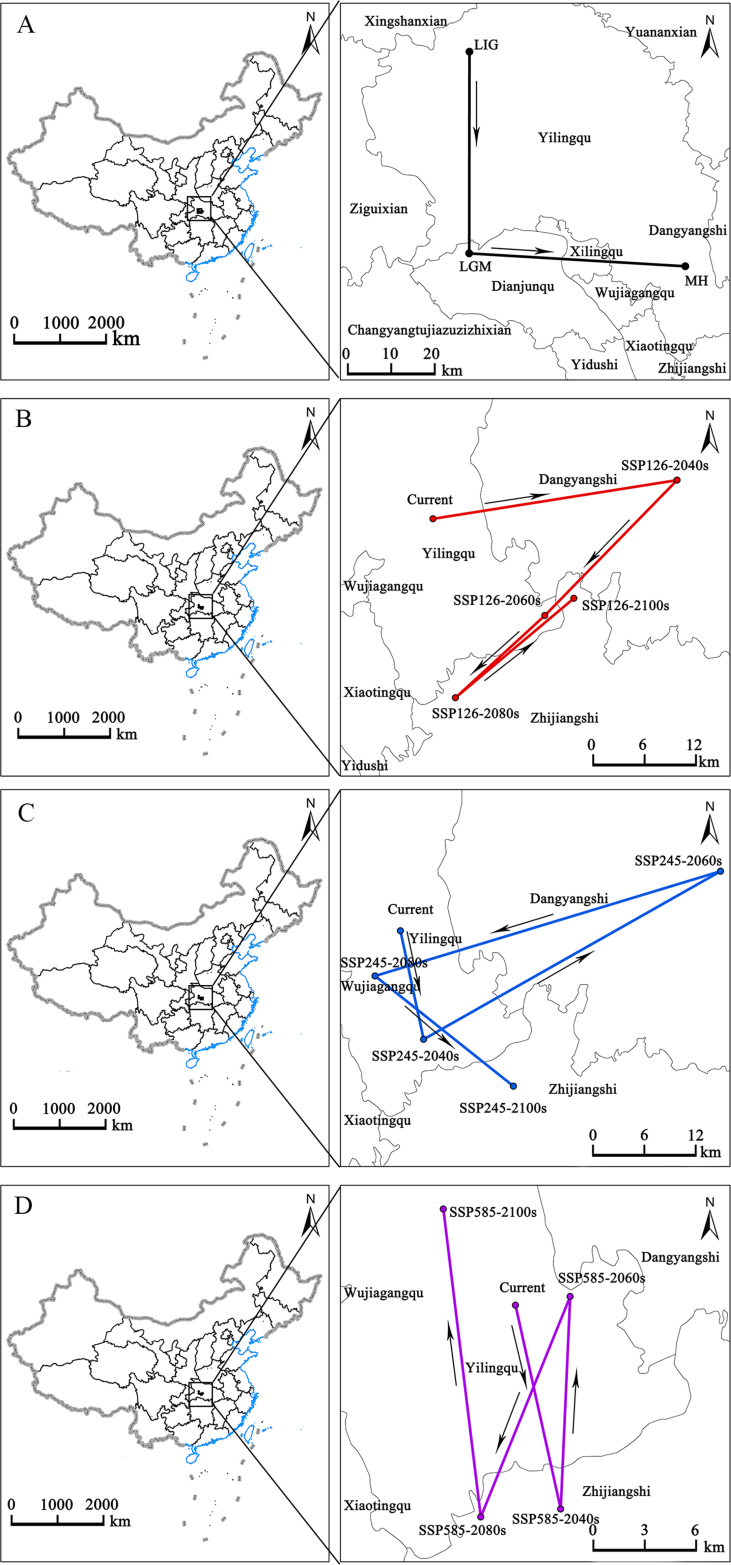
Migration of the center of suitable habitat for *E. ulmoides* under different climate scenario/year. Arrows indicate the directions in which the centers of mass migrate in different time periods. From LIG to MH **(A)**, under the SSP126 **(B)**, under the SSP245 **(C)**, under the SSP245 **(C)**, under the SSP585 **(D)**.

The geometric center was used in this study to represent the overall spatial position of potentially suitable *E. ulmoides* habitat since the last interglacial period (**Figure 11**). Currently, the geometric center of potentially suitable *E. ulmoides* habitat is located in Yiling District, Yichang City, Hubei Province. During the last interglacial period, the Last Glacial Maximum, and Mid-Holocene, the centers of *E. ulmoides* distributions were situated in Yiling District, Dianjun District, and Yiling District in Hubei Province, respectively. Under the SSP1-2.6 scenario, the center is projected to shift from Yiling District to Dangyang District by the 2040s, return to Yiling District by the 2060s, and move to Zhijiang District by the 2080s. Under the SSP2-4.5 scenario, the center will be in Yiling District by the 2040s, shift to Dangyang District by the 2060s, revert to Yiling District by the 2080s, and finally move to Zhijiang District by the 2100s. Under the SSP5-8.5 scenario, the center is expected to shift from Yiling District to Zhijiang District by the 2040s, revert to Yiling District by the 2060s, move to Zhijiang District by the 2080s, and finally return to Yiling District by the 2100s.

## 4 Discussion

### 4.1 Model predictive performance

Predicting the changes in *E. ulmoides* in response to climate change is crucial for protecting resources, introducing and cultivating, refining management, and sustainable utilization (Hamid et al., 2018). Employing the same dataset, different models yield varying predictions. Before finalizing the model, verifying the accuracy of various models is essential for obtaining reliable prediction results (Huang et al., 2023). First, we used 9 Biomod2 models for prediction. The results show that the AUC and TSS of the MaxEnt model are significantly greater than those of the other models, indicating that the MaxEnt model is more accurate. The results of our study are consistent with the results of previous studies, and the prediction effect of the MaxEnt model is better (Zhao et al., 2021a).

Subsequently, a MaxEnt model combined with ArcGIS was used to simulate the potentially suitable areas for *E. ulmoides* in China using 257 distribution points and 19 environmental variables. To minimize errors, an extensive parameter optimization process was conducted. This process tested eight numerical regularization multipliers ranging from 0.5 to 4 and nine feature combinations. As results, in a total 72 parameter combinations were evaluated using the ENMeval package in R software. The findings indicated that optimizing the MaxEnt model parameters (RM = 2.0) with FC = LQHPT yielded the best results, as evidenced by a decrease in the AICc value of 37.48, suggesting a reduction in overfitting after optimization. Thus, employing optimized parameters within the MaxEnt model can effectively reduce overfitting problems while enhancing the predictions of species distribution.

### 4.2 Effects of the main environmental variables on the distribution of *E. ulmoides*

Environmental factors are often the most critical parameters that determine the growth and development of an organism, as well as the geographic distribution, diversity, and evolution of species (Zhao et al., 2021a, b). The analysis using the MaxEnt model indicated that the distribution of *E. ulmoides* in China is primarily influenced by several key factors, including the minimum temperature of the coldest month, annual mean temperature, annual precipitation, precipitation of the wettest month, temperature seasonality, and mean temperature of the coldest quarter. The environmental conditions suitable for the growth of *E. ulmoides* occur when the minimum temperature of the coldest month exceeds -5°C, the annual mean temperature is above 14°C, and the annual precipitation ranges between 900 mm and 1900 mm. The results demonstrated that precipitation and temperature are significant factors limiting the current distribution of *E. ulmoides*. Hydrothermal conditions are crucial in determining plant distribution, with terrestrial plants showing particular sensitivity to temperature and precipitation (Bradie et al., 2017). Temperature and precipitation influence a series of physiological activities during seed germination, growth, and development (Punyasena et al., 2010).

### 4.3 Changes in the potential geographical distribution of *E. ulmoides*

In China, *E. ulmoides* is predominantly found in regions characterized by a subtropical monsoon climate south of the Yangtze River, where hydrothermal resources are plentiful. The predictions from the MaxEnt model indicate that the potential distribution area for *E. ulmoides* is mainly concentrated in Chongqing, Guizhou, Guangxi, Hainan, Hunan, Jiangxi, Hubei, Anhui, Henan, Shandong Provinces, most of the provinces of Guangdong, Fujian, Jiangsu, eastern Sichuan Province, and parts of Shanxi and Shaanxi Provinces. Under future climate scenarios, the predicted potentially suitable areas for *E. ulmoides* are expected to expand, primarily to the north. Similar to the migration of this species to higher altitudes and latitudes (Grabherr et al., 1994; Zu et al., 2021), there is only a small range of migration observed for centroids, with some returning to their original region. Northern China, which experiences low annual rainfall and cold, dry winters, is deemed unsuitable or less suitable for *E. ulmoides.*

### 4.4 Priority protected areas of *E. ulmoides*

*E. ulmoides* has long been utilized as a medicinal resource in China. The industry surrounding *E. ulmoides* encompasses sectors such as daily chemicals, food, rubber, and materials (Zhu et al., 2020; Gao et al., 2023; Wei X. et al., 2021). The bark, leaves, seeds, and wood of *E. ulmoides* hold significant economic value (Zhu and Sun, 2018). There is a historical precedence of *E. ulmoides* cultivation in China to fulfill the rising demand for its products (Du and Tan, 1996). To counter the environmental impact of extensive *E. ulmoides* cultivation, it is essential to develop more sustainable cultivation practices that are informed by an enhanced understanding of the species’ potential distribution. The MaxEnt model has been utilized to forecast the highly suitable regional distribution of *E. ulmoides* in various regions, including Eastern Sichuan, Chongqing, Guizhou, Hunan, Guangxi, Guangdong, and Jiangxi. Most of these areas are warm and humid mountainous areas with good natural conditions, which are consistent with the growth habits of most medicinal plants and are very suitable for the growth of Chinese herbal medicines (Liu et al., 2021). These regions are likely to maintain stability under various climate change scenarios. The growth of *E. ulmoides* in these regions is expected to be minimally impacted by future climate warming. Therefore, these areas can be designated as priority conservation zones for *E. ulmoides*, where a substantial quantity of *E. ulmoides* can be cultivated.

## 5 Conclusions

In formulating natural resource management policies, accurate and reliable results are often required. In this study, Biomod2 platform was used to simulate the appropriate distribution of *E. ulmoides*. After screening 9 single models, MaxEnt model showed high accuracy and could predict the distribution and change of *E. ulmoides* resources well. Moreover the potential distribution map produced by the optimized MaxEnt model is closer to the actual distribution of *E. ulmoides*, so it can provide more accurate and valuable information. The MaxEnt model was used to ascertain the distribution of potentially suitable habitats for *E. ulmoides* across China under historical, current, and future climate conditions. The results indicate that the minimum temperature of the coldest month, the annual mean temperature, and the annual precipitation significantly influence the distribution of *E. ulmoides*. The most favorable habitats under the current climate conditions were found in the provinces of Chongqing, Guizhou, Guangxi, Guangdong, Hunan, Jiangxi, Fujian, Hubei, Anhui, Jiangsu, Henan, Shandong, and Hainan. The areas classified as highly, moderately, and minimally suitable for *E. ulmoides* covered 106.37 × 10^4^ km^2^, 124.80 × 10^4^ km^2^, and 77.22 × 10^4^ km^2^, respectively, with most highly suitable areas located in Southwest and Southeast China. In the future, under varying climate scenarios, these highly suitable regions are anticipated to remain relatively unchanged. This highlights their importance for the conservation of *E. ulmoides* germplasm. The Yichang region in Hubei Province is expected to become an important migratory hub for *E. ulmoides* under changing climatic conditions. Additionally, the suitable distribution range of *E. ulmoides* is predicted to expand northward in response to varying climate conditions. This study provides valuable empirical data to support the conservation and sustainable management of *E. ulmoides* resources in the future.

## Data Availability

The original contributions presented in the study are included in the article/**Supplementary Material**, further inquiries can be directed to the corresponding author/s.
