# Supplementary material for: Prediction of the potentially suitable areas of Eucommia ulmoides Oliver in China under climate change based on optimized Biomod2 and MaxEnt models
Source: Front Plant Sci. 2024 Nov 15;15:1359271. doi: 10.3389/fpls.2024.1359271 (PMC11604462; doi:10.3389/fpls.2024.1359271)
Supplement: Supplementary file 1 [file Table1.doc]

Supplementary Table S1. Longitude and latitude of 257 *E. ulmoides* points

| NO. | longitude | latitude | NO. | longitude | latitude |
| --- | --- | --- | --- | --- | --- |
| 1 | 100.1761271 | 26.83947452 | 131 | 110.0094604 | 24.83947452 |
| 2 | 101.5511271 | 21.46447452 | 132 | 110.0511271 | 21.88114119 |
| 3 | 101.6344604 | 34.71447452 | 133 | 110.0927938 | 29.38114119 |
| 4 | 101.9677938 | 24.08947452 | 134 | 110.1344604 | 26.58947452 |
| 5 | 102.0927938 | 26.92280785 | 135 | 110.1761271 | 24.13114119 |
| 6 | 102.3427938 | 29.21447452 | 136 | 110.1761271 | 36.88114119 |
| 7 | 102.3844604 | 24.17280785 | 137 | 110.2594604 | 21.25614119 |
| 8 | 102.4261271 | 28.29780785 | 138 | 110.2594604 | 26.88114119 |
| 9 | 102.7594604 | 28.96447452 | 139 | 110.2594604 | 28.21447452 |
| 10 | 102.8427938 | 29.79780785 | 140 | 110.3011271 | 33.67280785 |
| 11 | 103.0927938 | 30.08947452 | 141 | 110.3011271 | 37.63114119 |
| 12 | 103.2594604 | 27.71447452 | 142 | 110.3844604 | 19.63114119 |
| 13 | 103.3011271 | 26.38114119 | 143 | 110.3844604 | 23.54780785 |
| 14 | 103.6761271 | 22.96447452 | 144 | 110.4261271 | 31.00614119 |
| 15 | 103.9261271 | 30.75614119 | 145 | 110.6344604 | 24.63114119 |
| 16 | 104.2594604 | 31.25614119 | 146 | 110.6761271 | 31.71447452 |
| 17 | 104.3011271 | 28.83947452 | 147 | 110.7177938 | 34.33947452 |
| 18 | 104.4677938 | 25.71447452 | 148 | 110.9261271 | 22.38114119 |
| 19 | 104.4677938 | 30.58947452 | 149 | 110.9677938 | 25.04780785 |
| 20 | 104.6761271 | 31.75614119 | 150 | 110.9677938 | 31.58947452 |
| 21 | 104.7177938 | 27.17280785 | 151 | 111.0094604 | 27.13114119 |
| 22 | 104.8844604 | 27.42280785 | 152 | 111.0094604 | 34.04780785 |
| 23 | 104.9261271 | 28.58947452 | 153 | 111.0927938 | 23.75614119 |
| 24 | 105.0927938 | 31.08947452 | 154 | 111.0927938 | 25.46447452 |
| 25 | 105.1761271 | 25.42280785 | 155 | 111.1344604 | 29.67280785 |
| 26 | 105.2177938 | 28.29780785 | 156 | 111.2594604 | 31.92280785 |
| 27 | 105.2177938 | 32.58947452 | 157 | 111.3011271 | 26.42280785 |
| 28 | 105.3427938 | 24.79780785 | 158 | 111.3427938 | 38.00614119 |
| 29 | 105.3844604 | 26.33947452 | 159 | 111.4261271 | 30.38114119 |
| 30 | 105.3844604 | 29.13114119 | 160 | 111.4261271 | 34.08947452 |
| 31 | 105.4261271 | 33.79780785 | 161 | 111.5094604 | 23.21447452 |
| 32 | 105.5511271 | 30.50614119 | 162 | 111.5511271 | 25.54780785 |
| 33 | 105.5927938 | 23.63114119 | 163 | 111.6761271 | 27.67280785 |
| 34 | 105.6344604 | 27.13114119 | 164 | 111.6761271 | 33.63114119 |
| 35 | 105.6344604 | 29.38114119 | 165 | 111.8011271 | 27.21447452 |
| 36 | 105.6761271 | 25.21447452 | 166 | 112.1344604 | 24.75614119 |
| 37 | 105.6761271 | 28.58947452 | 167 | 112.1761271 | 25.58947452 |
| 38 | 105.6761271 | 33.29780785 | 168 | 112.1761271 | 35.71447452 |
| 39 | 105.7594604 | 26.08947452 | 169 | 112.2594604 | 33.00614119 |
| 40 | 105.7594604 | 33.00614119 | 170 | 112.4261271 | 34.33947452 |
| 41 | 105.8011271 | 23.42280785 | 171 | 112.5927938 | 24.46447452 |
| 42 | 105.8011271 | 28.04780785 | 172 | 112.8011271 | 23.96447452 |
| 43 | 106.0511271 | 27.00614119 | 173 | 113.0094604 | 28.21447452 |
| 44 | 106.0927938 | 30.46447452 | 174 | 113.2594604 | 23.04780785 |
| 45 | 106.1344604 | 25.75614119 | 175 | 113.2594604 | 24.75614119 |
| 46 | 106.1344604 | 28.17280785 | 176 | 113.3844604 | 25.33947452 |
| 47 | 106.1344604 | 33.33947452 | 177 | 113.5511271 | 28.71447452 |
| 48 | 106.2177938 | 24.29780785 | 178 | 113.6761271 | 25.54780785 |
| 49 | 106.2594604 | 26.42280785 | 179 | 113.7594604 | 24.29780785 |
| 50 | 106.3011271 | 25.29780785 | 180 | 113.8011271 | 35.25614119 |
| 51 | 106.3011271 | 32.21447452 | 181 | 113.9677938 | 29.17280785 |
| 52 | 106.4677938 | 29.46447452 | 182 | 114.0511271 | 32.17280785 |
| 53 | 106.5511271 | 27.58947452 | 183 | 114.1344604 | 27.46447452 |
| 54 | 106.5927938 | 23.33947452 | 184 | 114.1761271 | 38.04780785 |
| 55 | 106.6761271 | 26.96447452 | 185 | 114.2177938 | 26.96447452 |
| 56 | 106.7177938 | 33.13114119 | 186 | 114.2177938 | 34.21447452 |
| 57 | 106.8011271 | 28.13114119 | 187 | 114.3844604 | 28.50614119 |
| 58 | 106.8844604 | 22.33947452 | 188 | 114.3844604 | 30.88114119 |
| 59 | 106.9677938 | 26.46447452 | 189 | 114.4261271 | 25.79780785 |
| 60 | 106.9677938 | 27.08947452 | 190 | 114.4261271 | 36.17280785 |
| 61 | 107.0511271 | 24.38114119 | 191 | 114.5511271 | 26.29780785 |
| 62 | 107.0927938 | 29.17280785 | 192 | 114.5511271 | 29.04780785 |
| 63 | 107.1344604 | 23.08947452 | 193 | 114.5927938 | 33.63114119 |
| 64 | 107.1344604 | 32.58947452 | 194 | 114.6761271 | 27.83947452 |
| 65 | 107.2594604 | 31.92280785 | 195 | 114.8427938 | 31.63114119 |
| 66 | 107.3011271 | 28.58947452 | 196 | 114.9261271 | 24.46447452 |
| 67 | 107.3011271 | 33.13114119 | 197 | 114.9261271 | 28.83947452 |
| 68 | 107.3427938 | 22.38114119 | 198 | 115.0927938 | 29.29780785 |
| 69 | 107.3427938 | 25.83947452 | 199 | 115.1761271 | 27.21447452 |
| 70 | 107.3427938 | 30.33947452 | 200 | 115.2594604 | 25.71447452 |
| 71 | 107.3844604 | 24.50614119 | 201 | 115.3011271 | 28.88114119 |
| 72 | 107.4261271 | 28.17280785 | 202 | 115.3844604 | 30.83947452 |
| 73 | 107.5094604 | 26.25614119 | 203 | 115.5094604 | 34.71447452 |
| 74 | 107.5094604 | 27.75614119 | 204 | 115.8844604 | 24.92280785 |
| 75 | 107.5511271 | 22.88114119 | 205 | 116.0511271 | 40.38114119 |
| 76 | 107.5511271 | 24.96447452 | 206 | 116.0927938 | 34.83947452 |
| 77 | 107.7177938 | 34.13114119 | 207 | 116.1761271 | 27.54780785 |
| 78 | 107.7594604 | 33.00614119 | 208 | 116.2177938 | 26.88114119 |
| 79 | 107.8427938 | 27.21447452 | 209 | 116.2177938 | 31.21447452 |
| 80 | 107.8844604 | 25.42280785 | 210 | 116.3427938 | 39.92280785 |
| 81 | 107.8844604 | 28.54780785 | 211 | 116.5927938 | 29.67280785 |
| 82 | 107.9261271 | 22.63114119 | 212 | 116.6344604 | 24.13114119 |
| 83 | 107.9677938 | 33.67280785 | 213 | 116.6761271 | 36.33947452 |
| 84 | 108.0511271 | 26.67280785 | 214 | 116.8011271 | 26.17280785 |
| 85 | 108.0511271 | 32.08947452 | 215 | 116.8011271 | 35.75614119 |
| 86 | 108.0927938 | 30.00614119 | 216 | 116.8844604 | 27.29780785 |
| 87 | 108.1344604 | 27.04780785 | 217 | 117.0927938 | 36.71447452 |
| 88 | 108.1344604 | 29.29780785 | 218 | 117.1761271 | 35.08947452 |
| 89 | 108.2177938 | 26.29780785 | 219 | 117.2177938 | 23.75614119 |
| 90 | 108.2594604 | 23.13114119 | 220 | 117.2594604 | 35.67280785 |
| 91 | 108.3011271 | 24.83947452 | 221 | 117.3011271 | 40.21447452 |
| 92 | 108.3011271 | 31.08947452 | 222 | 117.3427938 | 24.50614119 |
| 93 | 108.3427938 | 21.75614119 | 223 | 117.5511271 | 30.21447452 |
| 94 | 108.3844604 | 27.96447452 | 224 | 117.6761271 | 36.17280785 |
| 95 | 108.4677938 | 26.71447452 | 225 | 117.7177938 | 34.63114119 |
| 96 | 108.5094604 | 27.50614119 | 226 | 117.8427938 | 26.83947452 |
| 97 | 108.5094604 | 32.88114119 | 227 | 117.8427938 | 36.83947452 |
| 98 | 108.5927938 | 23.42280785 | 228 | 117.8427938 | 40.88114119 |
| 99 | 108.5927938 | 28.50614119 | 229 | 117.9261271 | 35.67280785 |
| 100 | 108.5927938 | 34.00614119 | 230 | 118.1761271 | 30.13114119 |
| 101 | 108.6344604 | 24.04780785 | 231 | 118.2594604 | 32.29780785 |
| 102 | 108.6761271 | 30.92280785 | 232 | 118.3844604 | 37.04780785 |
| 103 | 108.7594604 | 27.21447452 | 233 | 118.4261271 | 29.13114119 |
| 104 | 108.7594604 | 29.54780785 | 234 | 118.4677938 | 36.50614119 |
| 105 | 108.9261271 | 24.75614119 | 235 | 118.5094604 | 30.50614119 |
| 106 | 108.9261271 | 30.29780785 | 236 | 118.5511271 | 39.92280785 |
| 107 | 108.9261271 | 32.29780785 | 237 | 118.6344604 | 35.79780785 |
| 108 | 109.0094604 | 28.54780785 | 238 | 118.8011271 | 36.71447452 |
| 109 | 109.0511271 | 25.88114119 | 239 | 118.8844604 | 42.25614119 |
| 110 | 109.1761271 | 18.75614119 | 240 | 119.1761271 | 35.71447452 |
| 111 | 109.1761271 | 21.67280785 | 241 | 119.2594604 | 42.04780785 |
| 112 | 109.1761271 | 32.58947452 | 242 | 119.3427938 | 29.46447452 |
| 113 | 109.3011271 | 22.42280785 | 243 | 119.3844604 | 30.79780785 |
| 114 | 109.3011271 | 29.88114119 | 244 | 119.4261271 | 32.17280785 |
| 115 | 109.3427938 | 28.71447452 | 245 | 119.5511271 | 34.21447452 |
| 116 | 109.3844604 | 30.38114119 | 246 | 119.6761271 | 31.21447452 |
| 117 | 109.4261271 | 19.21447452 | 247 | 119.8844604 | 36.79780785 |
| 118 | 109.4261271 | 31.00614119 | 248 | 120.4261271 | 37.33947452 |
| 119 | 109.4677938 | 28.25614119 | 249 | 120.6761271 | 31.63114119 |
| 120 | 109.5927938 | 31.42280785 | 250 | 120.8844604 | 40.75614119 |
| 121 | 109.6344604 | 23.04780785 | 251 | 120.9261271 | 36.79780785 |
| 122 | 109.6344604 | 27.00614119 | 252 | 121.5511271 | 29.88114119 |
| 123 | 109.6761271 | 37.13114119 | 253 | 121.6761271 | 42.00614119 |
| 124 | 109.7177938 | 25.92280785 | 254 | 122.0094604 | 39.71447452 |
| 125 | 109.7177938 | 32.33947452 | 255 | 123.6344604 | 41.92280785 |
| 126 | 109.7594604 | 30.25614119 | 256 | 98.88446045 | 26.92280785 |
| 127 | 109.8011271 | 19.08947452 | 257 | 99.09279378 | 30.00614119 |
| 128 | 109.8011271 | 34.46447452 |  |  |  |
| 129 | 109.9677938 | 29.00614119 |  |  |  |
| 130 | 110.0094604 | 18.46447452 |  |  |  |

**Supplementary Table S2. 19 bioclimatic variables information in this study**

| Variable | Description | Unit |
| --- | --- | --- |
| bio1 | Annual mean temperature | °C |
| bio2 | Mean diurnal range (Mean of monthly (max.temp.-min.temp.) | °C |
| bio3 | Isothermality (bio2 / bio7) (× 100) | - |
| bio4 | Temperature seasonality (standard deviation×100) | - |
| bio5 | Max temperature of the warmest month | °C |
| bio6 | Min temperature of the coldest month | °C |
| bio7 | Temperature annual range (bio5- bio6) | °C |
| bio8 | Mean temperature of the wettest quarter | °C |
| bio9 | Mean temperature of the driest quarter | °C |
| bio10 | Mean temperature of the warmest quarter | °C |
| bio11 | Mean temperature of the coldest quarter | °C |
| bio12 | Annual precipitation | mm |
| bio13 | Precipitation of the wettest month | mm |
| bio14 | Precipitation of the driest month | mm |
| bio15 | Precipitation seasonality (Coefficient of variation) | - |
| bio16 | Precipitation of the wettest quarter | mm |
| bio17 | Precipitation of the driest quarter | mm |
| bio18 | Precipitation of the warmest quarter | mm |
| bio19 | Precipitation of coldest quarter | mm |
